# Hypoalbuminemia and Obesity in Orthopaedic Trauma Patients: Body Mass Index a Significant Predictor of Surgical Site Complications

**DOI:** 10.1038/s41598-020-58987-4

**Published:** 2020-02-06

**Authors:** Ryan C. Egbert, Trevor T. Bouck, Nikhil N. Gupte, Miren M. Pena, Khang H. Dang, Samuel S. Ornell, Boris A. Zelle

**Affiliations:** The University of Texas Health Science Center at San Antonio, Department of Orthopedics, San Antonio, Texas 78229 USA

**Keywords:** Prognostic markers, Outcomes research, Comorbidities

## Abstract

The purpose of this investigation was to identify the prevalence of hypoalbuminemia and obesity in orthopaedic trauma patients with high-energy injuries and to investigate their impact on the incidence of surgical site complications. Patients 18 years of age and older undergoing intramedullary nail fixation of their femoral shaft fractures at a university-based level-1 trauma centre were assessed. Malnutrition was measured using serum markers (albumin <3.5 g/dL) as well as body mass index (BMI) as a marker of obesity (BMI > 30 kg/m^2^). The primary outcome measure was surgical wound complications. A total of 249 patients were included in this study. Ninety-eight patients (39.4%) presented with hypoalbuminaemia and 80 patients (32.1%) were obese. The overall incidence of wound complications in our study population was 9.65% (n = 25/259). A logistic regression model showed that non-obese patients (BMI < 30 kg/m^2^) were at significantly reduced risk for perioperative wound complications (Odds Ratio 0.400 [95% confidence interval 0.168, 0.954], p = 0.039). This study demonstrated a substantial prevalence of hypoalbuminemia and obesity among orthopaedic trauma patients with high-energy injuries. Obesity may increase the risk of surgical site complications. Future studies are required to further define malnutrition and its correlation with surgical site complications in orthopaedic trauma patients.

## Introduction

Malnutrition represents a significant healthcare problem and complicates medical and surgical patient care^1–9^. According to the American Society for Parenteral and Enteral Nutrition (ASPEN), malnutrition is defined as a state of nutrition, in which a combination of varying degrees of overnutrition or undernutrition have led to changes in body composition and diminished function (www.nutritioncare.org, last accessed December 25, 2019). Data from the medicine literature have suggested that malnutrition may negatively affect the treatment outcomes of various medical conditions, such as cerebrovascular disease, heart disease, pulmonary disease, renal failure, diabetes, and cancer^1–6^. In addition, investigations in the field of general surgery have reported malnutrition as a significant risk factor for patient morbidity and mortality^7–9^. Within the orthopaedic surgery literature, the preoperative nutritional status as a predictor of surgical outcomes has recently gained attention^10–15^. In particular, for elective orthopaedic procedures, malnutrition has been reported as a significant risk factor for surgical complications. Thus, it has been reported that malnourished patients undergoing elective hip or knee arthroplasty will be at significantly higher risk for surgical complications^10–12^. Similarly, recent reports have suggested that malnourished patients undergoing elective spinal fusion are at significantly higher risk for increased length of stay, hospital readmission, surgical complications, and mortality^13–15^. Regarding surgical complications, particularly wound healing problems and infections have been emphasized to be associated with a poor nutritional status.

Data from the literature about malnutrition in orthopaedic trauma patients remains limited. Most investigations in the field of orthopaedic trauma address nutritional deficits in elderly patients undergoing hip fracture surgery^16–18^. However, reports on young orthopaedic trauma patients with high-energy injuries remain limited. Therefore, the goals of this study are (1) to identify the prevalence of hypoalbuminemia and obesity in orthopaedic trauma patients with high-energy injuries and (2) to investigate their impact on the incidence of surgical site complications. We hypothesise that hypoalbuminemia and obesity increase the risk of surgical site complications in orthopaedic trauma patients with high-energy injuries.

## Materials and Methods

This was a retrospective study that was performed at an urban university-based level-1 trauma centre. The study data was collected through a retrospective chart review and review of existing laboratory and radiographic studies. Patients were identified through the coding database of our institution. Approval of the study protocol was obtained from the Institutional Review Board (IRB) of our institution and all methods were performed in accordance with the relevant guidelines and regulations. As per institutional IRB regulations, this investigation was exempt from requiring patient consent for study participation since this investigation did not include any patient intervention or direct patient contact. Thus, the need for informed consent was waived by our institutional IRB. Only investigators listed on the approved IRB protocol had access to identifying patient information.

Orthopaedic trauma patients 18 years of age and older undergoing intramedullary nail fixation of their femoral fractures between 2013 and 2016 were included in this investigation. Sensitive patient populations, such as prisoners, patients with mental disorders, and pregnant patients were excluded from this study. In addition, patients with pathologic fractures were excluded from this investigation.

All patients included in this study underwent intramedullary nail fixation of their femoral shaft fractures. The albumin levels were retrieved from the patient charts as they are obtained routinely as part of the standard admission laboratory studies in trauma patients at our institution. We also collected demographic and clinical data, such as age, gender, body mass index (BMI), medical co-morbidities, smoking history, insurance coverage, employment status, race/ethnicity, American Society of Anesthesiologists’ (ASA) score^19^, mechanism of injury, and fracture type (open versus closed). In addition, clinical data on the hospital course were collected from the patient charts, including injury severity score (ISS)^20^, intensive care unit days, ventilator days, and length of hospital stay. Hypoalbuminaemia was defined as a serum albumin level of less than 3.5 g/dL. Obesity was defined as BMI > 30 kg/m^2^. The incidence of wound complications, including wound dehiscence and wound infection, was retrieved from the inpatient and outpatient electronic medical records.

### Statistical analysis

The statistical analyses were performed on a total of 249 patients who were identified by the inclusion and exclusion criteria as above. All statistical analysis was performed using Stata 15.1 (StataCorp, College Station, TX). Binary variables were compared using the chi-square test. Continuous variables were compared using the Wilcoxon test. A logistic regression model was created in order to identify independent variables predicting the incidence of wound complications. The independent variables tested included age, gender, diabetes, smoking, race/ethnicity, BMI, insurance type, ASA score, illicit drug abuse, hypoalbuminaemia, open fracture, and mechanism of injury. The dependent binary variable was incidence of wound complications. First, a univariate analysis was performed to identify independent variables that demonstrated a significant difference for the incidence of wound complications. All independent variables that showed a significant difference (p < 0.05) or trended differently (p = 0.05–0.15) between patients with and without wound complications, respectively were included in the logistic regression model. Thus, the independent variables tested in our logistic regression model included BMI, insurance type, ASA score, and mechanism of injury.

## Results

### Cohort characteristics

**Figure 1:**
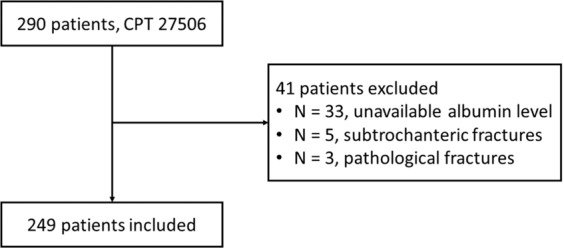
Flowchart of study population.

Based on the Current Procedural Terminology (CPT) code 27506, a total of 290 patients were screened for participation in this study. Forty-one patients did not meet the inclusion criteria. The specific reasons for exclusion from the study were unavailable albumin level (n = 33), subtrochanteric fracture (n = 5), and pathological fracture (n = 3). The outcome data reported herein are based on 249 patients (Fig. 1).

The mean age of the 249 patients included in this study was 34.4 years old (SD 16.5) (Table 1). A total of 185 patients (74.3%) were male and 64 patients (25.7%) were female. Twenty patients (8.0%) were recorded to have diabetes. An ASA score of 1 or 2 was recorded for 126 patients (50.6%) patients, while 123 patients (49.4%) had an ASA score of 3 or 4. In regards to the ethnicity, 151 patients (60.6%) were recorded as Hispanics, and 98 patients (39.4%) were non-Hispanic. A total of 80 patients (32.1%) were recorded as obese with a BMI greater than or equal to 30 kg/m^2^. In addition, 117 patients (47.0%) were recorded as smokers, and 127 patients (51.0%) had a history of illicit drug abuse. The mean length of hospital stay was 9.44 days (range 1–70). A total of 97 patients (40%) required time in the ICU (mean 9.18 days, range 1–45). Sixty patients (24.1%) required a ventilator for a mean of 8.26 days (range 1–31).

**Table 1:** Characteristics of patients with and without hypoalbuminemia.

Patient Characteristic	Albumin Level < 3.5 (n = 98)	Albumin Level ≥ 3.5 (n = 151)	Total	P-Value
Age, mean ± SD, y	39.4 ± 18.3	31.2 ± 14.4	34.4 ± 16.5	<0.001*
Gender, n (%)	—	—	—	0.043*
*Male*	66 (35.7%)	119 (64.3%)	185 (74.3%)	
*Female*	32 (50.0%)	32 (50.0%)	64 (25.7%)	
Diabetes, n (%)	—	—	—	0.049*
*Yes*	12 (60.0%)	8 (40.0%)	20 (8.0%)	
*No*	86 (37.6%)	143 (62.4%)	229 (92.0%)	
Smoking, n (%)	—	—	—	0.036*
*Yes*	38 (32.5%)	79 (67.5%)	117 (47.0%)	
*No*	60 (45.5%)	72 (54.5%)	132 (53.0%)	
Ethnicity, n (%)	—	—	—	0.909
*Hispanic*	59 (39.1%)	92 (60.9%)	151 (60.6%)	
*Non-Hispanic*	39 (39.8%)	59 (60.2%)	98 (39.4%)	
BMI, n (%)	—	—	—	0.126
*BMI* < 30	61 (36.1%)	108 (63.9%)	169 (67.9%)	
*BMI* ≥ 30	37 (46.3%)	43 (53.7%)	80 (32.1%)	
Insurance Type, n (%)	—	—	—	0.186
*Self-Pay*	29 (33.7%)	57 (66.3%)	86 (34.5%)	
*Insurance/Other*	69 (42.3%)	94 (57.7%)	163 (65.5%)	
ASA Classification^#^, n (%)	—	—	—	<0.001*
*1 or 2*	26 (20.6%)	100 (79.4%)	126 (50.6%)	
*3 or 4*	72 (58.5%)	51 (41.5%)	123 (50.4%)	
Illicit Drug Use^¥^, n (%)	—	—	—	0.264
*Yes*	37 (35.9%)	66 (64.1%)	103 (45.2%)	
*No*	54 (43.2%)	71 (56.8%)	125 (54.8%)	
Open Fracture, n (%)	—	—	—	<0.001*
*Yes*	31 (60.8%)	20 (39.2%)	51 (20.1%)	
*No*	67 (33.8%)	131 (66.2%)	198 (79.9%)	

^#^No subjects exhibited an ASA score of 5.

^¥^Data not available for all subjects (n = 228).

*P-value significant at 0.05 level.

### Hypoalbuminemia and obesity

Overall, 98 patients (39.4%) were recorded to have hypoalbuminaemia (Table 1). The rate of hypoalbuminaemia was significantly increased in female patients (50.0% versus 35.7% in male patients, p = 0.043), patients with diabetes (60.0% versus 37.6% in non-diabetic patients, p = 0.049), patients with an ASA score of 3 or 4 (73.5% versus 26.5% in patients with an ASA of 1 or 2, p < 0.001). Additionally, patients with hypoalbuminaemia were significantly older than patients with normal albumin levels (mean 39.3 ± SD 18.3 versus 31.1 ± 14.4, p = 0.008).

Obese patients showed a statistically non-significant trend towards being older than non-obese patients 36.6 ± 16.2 versus 33.4 ± 16.6, p = 0.150) (Table 2). The rate of obesity was higher in patients with an ASA score of 3 or 4 as compared to an ASA score of 1 or 2 (p = 0.010), whereas patients with a history of illicit drug abuse were at significantly lower risk for obesity (p = 0.015). The rate of obesity trended higher in Hispanic patients as compared to non-Hispanics, but this was not statistically significant (p = 0.128).

**Table 2:** Characteristics of non-obese vs. obese patients.

Patient Characteristic	Non-Obese (BMI < 30) (n = 169)	Obese (BMI ≥ 30) (n = 80)	Total	P-Value
Age, mean ± SD, y	33.4 ± 16.6	36.6 ± 16.2	34.4 ± 16.5	0.150
Gender, n (%)	—	—	—	0.628
*Male*	124 (73.4%)	61 (76.3%)	185 (74.3%)	
*Female*	45 (26.6%)	19 (21.2%)	64 (23.7%)	
Diabetes, n (%)	—	—	—	0.199
*Yes*	11 (7.1%)	9 (10.0%)	20 (8.0%)	
*No*	158 (92.9%)	71 (90.0%)	229 (92.0%)	
Smoking, n (%)	—	—	—	0.665
*Yes*	81 (47.9%)	36 (45.0%)	117 (47.0%)	
*No*	88 (52.1%)	44 (55.0%)	132 (53.0%)	
Ethnicity, n (%)	—	—	—	0.128
*Hispanic*	97 (57.4%)	54 (67.5%)	151 (60.6%)	
*Non-Hispanic*	72 (42.6%)	26 (32.5%)	98 (39.4%)	
Insurance Type, n (%)	—	—	—	0.916
*Self-Pay*	58 (34.3%)	28 (35.0%)	86 (34.5%)	
*Insurance/Other*	111 (65.7%)	52 (65.0%)	163 (65.5%)	
ASA Classification^#^, n (%)	—	—	—	0.010*
*1 or 2*	95 (56.2%)	31 (38.7%)	126 (50.6%)	
*3 or 4*	74 (43.8%)	49 (61.3%)	123 (50.4%)	
Illicit Drug Use, n (%)	—	—	—	0.015*
*Yes*	79 (55.0%)	24 (36.2%)	103 (51.0%)	
*No*	77 (45.0%)	48 (63.8%)	125 (49.0%)	
Open Fracture, n (%)	—	—	—	0.983
*Yes*	35 (20.1%)	16 (20.0%)	51 (20.1%)	
*No*	134 (79.9%)	64 (80.0%)	198 (79.9%)	

^#^No subjects exhibited an ASA score of 5.

^¥^Data not available for all subjects (n = 247).

*P-value significant at 0.05 level.

### Incidence of wound complications

The overall incidence of wound complications in our study population was 9.65% (n = 25/259) (Table 3). The rate of wound complications was significantly higher in obese patients as compared to non-obese patients (16.7% versus 7.1%, p = 0.021). In addition, insurance type, ASA, and mechanism of injury trended differently between patients with and without wound complications, respectively (p = 0.05–0.15). Therefore, BMI, insurance type, ASA score, and mechanism of injury represented the independent variables included in the logistic regression model (Table 4). Within the logistic regression model, BMI demonstrated a significantly increased the risk of wound complications (p = 0.039).

**Table 3:** Univariate analysis for patients with and without wound complications.

Patient Characteristic	No Wound Complication(s)^¥^ (n = 222)	Wound Complication(s)^¥^ (n = 25)	Total	P-Value
Gender, n (%)	—	—	—	0.724
*Male*	167 (90.3%)	18 (9.7%)	185 (74.9%)	
*Female*	55 (88.7%)	7 (11.3%)	62 (25.1%)	
Age, mean ± SD (%), y	34.1 ± 16.1	36.0 ± 18.9	34.4 ± 16.5	0.631
Diabetes, n (%)	—	—	—	0.985
*Yes*	18 (90.0%)	2 (10.0%)	20 (9.1%)	
*No*	204 (89.9%)	23 (9.1%)	227 (91.9%)	
Smoking, n (%)	—	—	—	0.362
*Yes*	103 (88.0%)	14 (12.0%)	117 (47.4%)	
*No*	119 (91.5%)	11 (8.5%)	130 (52.6%)	
Ethnicity, n (%)	—	—	—	0.756
*Hispanic*	135 (89.4%)	16 (10.6%)	151 (61.1%)	
*Non-Hispanic*	87 (90.6%)	9 (9.4%)	96 (38.9%)	
BMI, n (%)	—		—	0.021*
*BMI* < 30	157 (92.9%)	12 (7.1%)	169 (68.4%)	
*BMI* ≥ 30	65 (83.3%)	13 (16.7%)	78 (31.6%)	
Insurance Type, n (%)	—	—	—	0.144
*Self-Pay*	74 (86.0%)	12 (14.0%)	86 (34.8%)	
*Insurance/Other*	148 (91.9%)	13 (8.1%)	161 (65.2%)	
ASA Classification, n (%)	—	—	—	0.113
*1 or 2*	117 (92.8%)	9 (7.2%)	126 (51.0%)	
*3 or 4*	105 (86.8%)	16 (13.2%)	121 (49.0%)	
Illicit Drug Use^¥^, n (%)	—	—	—	0.645
*Yes*	91 (88.3%)	12 (11.7%)	103 (45.6%)	
*No*	111 (90.2%)	12 (9.8%)	123 (54.4%)	
Albumin Level, n (%)	—	—	—	0.323
*Albumin* < *3.5*	84 (87.5%)	12 (12.5%)	96 (38.9%)	
*Albumin* ≥ *3.5*	138 (91.4%)	13 (8.6%)	151 (61.1%)	
Open Fracture	—	—	—	0.662
*Yes*	45 (88.2%)	6 (11.8%)	51 (20.7%)	
*No*	177 (90.3%)	19 (9.7%)	196 (79.3%)	
Mechanism of Injury	—	—	—	0.076
*Motor vehicle crash, n* (%)	130 (92.8%)	10 (7.2%)	140 (56.7%)	
*Other, n* (%)	92 (86.0%)	15 (14.0%)	107 (43.3%)	

^#^No subjects exhibited an ASA score of 5.

^¥^Data not available for all subjects.

*P-value significant at 0.05 level.

**Table 4:** Logistic regression for incidence of wound complications.

Patient Characteristic	Odds Ratio (95% CI)	P-Value
BMI, n (%)	0.400 (0.168–0.954)	0.039*
Insurance Type	0.422 (0.174–1.024)	0.056
ASA Classification	0.507 (0.207–1.241)	0.137
Mechanism of Injury	2.652 (1.086–6.480)	0.032*

## Discussion

Malnutrition as a risk factor for poor outcomes has been well established in the medicine literature^1–6^ as well as the general surgery literature^7–9^. Within the field of orthopaedic surgery, hypoalbuminemia and obesity have been reported as significant risk factor for adverse outcomes in elective procedures, such as arthroplasty and spinal fusions^10–15^,. Data from the literature on malnutrition in orthopaedic trauma patients remains scarce and is mostly limited to studies in elderly patients undergoing hip fracture surgery^16–18^. Our study demonstrated that malnutrition represents a significant challenge in orthopaedic trauma patients with a hypoalbuminaemia rate of 39.4% and an obesity rate of 32.1%. Moreover, we recorded that obesity significantly increases the risk of surgical site complications in patients undergoing surgical fixation of their femur fracture.

Our study has several limitations. The data in our study was retrieved through a retrospective review. This type of data collection is inherently associated with certain limitations, arising from variable data entry into the electronic medical records. In addition, the available data on the nutritional status of patients included in this series was limited to albumin levels and BMI. Albumin has been widely established as a standard nutritional serum marker and has been used in numerous studies within the medicine, general surgery, and orthopaedic literature^2,3,5,9–16^. However, our study does not provide any information on clinical nutrition assessment scales or other nutritional serum markers that may be relevant to orthopaedic patients, such as micronutrients. Moreover, our data is limited to patients with femoral shaft fractures. A femur fracture model was chosen for the purpose of this study as we intended to capture a young orthopaedic trauma patient population with high-energy injuries. As femoral shaft fractures are typically the result of a high-energy injury, we feel that our study population appropriately represents this group of patients. However, one must be cautious when extrapolating our data to patient populations with other injury profiles. Our study identified obesity as an independent risk factor for wound complications. However, we also acknowledge that due to the relatively small sample size, our study may be underpowered to detect other significant risk factors for surgical site complications. Finally, the data recorded in this study reflects a single-institution experience. Thus, it must be assumed that prevalence and type of malnutrition is subject to geographic variations.

Comparing and contrasting our findings with data from the literature is challenging given the paucity of nutrition studies in this specific patient population. Ihle *et al*.^21^ reported the prevalence of malnutrition to be 22.3% in patients admitted to a German trauma center. Their patient population included both traumatic as well as elective admissions and patients were classified as malnourished using a clinical nutrition assessment scale. Regarding risk factors for malnutrition, our study identified age, female gender, diabetes, smoking, and ASA class 3 o r4 as risk factors for hypoalbuminaemia. This is consistent with previous investigations. Bohl *et al*.^22^ who reported hypoalbuminaemia to be associated with female gender as well as diabetes. In patients undergoing elective total joint arthroplasty. Furthermore, our findings are in accordance with previous investigations in patients with hip fractures which recorded an association between malnutrition and medical co-morbidities^23^. Lastly, poor nutritional status was significantly correlated with older age in our study, which is in line with Ihle *et al*., who also found their elderly patients to have a higher prevalence of malnutrition^21^. Our study found that obesity was a significant predictor of surgical site complications in orthopaedic trauma patients with high-energy injuries. This is in line with the experience of elective orthopaedic procedures that have reported similar results^10–15^. Our data emphasizes the urgent need for proper assessment of malnutrition in orthopaedic trauma patients during the early treatment phase.

## Conclusions

In conclusion, we recorded a substantial prevalence of hypoalbuminemia and obesity among orthopaedic trauma patients with high-energy injuries. In our patient population, obesity was found to be independent risk factor for surgical site complications. Our study emphasizes the necessity of proper assessment for malnutrition in orthopaedic trauma patients. Particularly, our study lays the ground for subsequent prospective investigations in this field. Future studies are required to further define the prevalence and the impact of malnutrition in other geographic regions. In addition, the role of other specific nutritional serum markers, such as micronutrients, must be established in future clinical investigations. In the long-term, this body of knowledge will provide healthcare providers with important tools potentially allowing for the development of cost efficient perioperative nutritional interventions that may minimize the risk of costly surgical site complications.

### Ethics approval

This study was approved by the Institutional Review Board of University of Texas Health at San Antonio.

## Data Availability

The datasets generated and/or analyzed during the current study are not publicly available due the presence of personal health information but are available from the corresponding author on reasonable request.
